# Increase in presentations with new-onset psychiatric disorders in a psychiatric emergency department in Berlin, Germany during the second wave of the COVID-19 pandemic – a retrospective cross-sectional study

**DOI:** 10.3389/fpsyt.2023.1240703

**Published:** 2023-10-12

**Authors:** Maia Adam, James K. Moran, Yann David Kippe, Meryam Schouler-Ocak, Felix Bermpohl, Stefan Gutwinski, Thomas Goldschmidt

**Affiliations:** Psychiatrische Universitätsklinik der Charité im St. Hedwig Krankenhaus, Berlin, Germany

**Keywords:** COVID-19, mental health, psychiatric disorder, new-onset, lockdown, psychiatric emergency department

## Abstract

**Introduction:**

While numerous studies have identified an increase in symptoms of depression as well as anxiety and distress due to the COVID-19 pandemic, relatively few studies have investigated the new-onset of psychiatric diseases during the pandemic.

**Methods:**

This study focuses on the number of psychiatric new-onset diagnoses in a psychiatric emergency department (pED) in Berlin, Germany during the second wave of the pandemic (i.e. from 09/15/2020 to 03/01/2021 = COVID-19-period) compared to pre-pandemic times (09/15/2019 to 03/01/2020 = control period). We focused on diagnostic subgroups and performed logistic regression analysis to investigate potential risk groups based on covariables such as age, gender, homelessness, attending in police custody and familial relationship.

**Results:**

Overall, there was a 59.7% increase in new-onset psychiatric diagnoses during the COVID-19-period. Increases in the following diagnoses were observed: new-onset of substance-related and addictive disorders (+192.5%), depressive disorders (+115.8%), schizophrenia spectrum and psychotic disorders (+113.3%) and anxiety disorders (+63.6%). These diagnostic subgroups, together with attending in police custody, were found to predict pED presentations with new-onset during the COVID-19-period. Interestingly, in the group of new-onset psychiatric diseases in the COVID-19-period, higher amounts of job loss and living alone as well as a relative decrease in familial relationships were observed.

**Discussion:**

COVID-19 infections and post-COVID-19 syndrome are unlikely to have played a substantial role in the increase of new-onset diseases in this study. Conclusion: Our findings underline the role of indirect factors in new-onset of psychiatric diseases during the pandemic and should be a caveat for future pandemic control policies.

## 1. Introduction

During the global COVID-19 pandemic, first recognized in December 2019, people were exposed to the acute health risks of COVID-19 infection (1), the potential long-term consequences of infection (2, 3), and a health system on the verge of collapse. Measurements such as travel restrictions, the closure of schools and workplaces, contact limitation, quarantine, isolation and also prevention of access to public places were implemented to mitigate the spread of the COVID-19 virus (4, 5).

The impact of the pandemic on mental health has been studied thoroughly already. Studies based on online questionnaires suggest a deterioration in the general population’s mental health since the beginning of the pandemic (6–9), predominantly regarding symptoms of depression, anxiety, insomnia, and acute stress. Most studies, however, show a substantial decline in psychiatric Emergency Department (pED) presentations (10–13), especially at the beginning of the pandemic. Yet, there are indicators that the presentations were more severe (14–16). Reasons for the decline might include the fear of getting infected with the COVID-19 virus in a pED (17–19), qualms about overloading the health system (20) or the government’s appeal to stay at home (21). An increase in the prevalence of psychiatric diseases might also lead to a deterioration of preexisting somatic diseases, as for example diabetes (22).

A global study from 2019 showed that mental disorders were among the leading cause of disability (16% of disability-adjusted life-years) in the last 20 years (23, 24). It can be assumed that if there was a significant increase in new-onset psychiatric disorders during the COVID-19 pandemic, this may lead to a simultaneous increase in the burden of psychiatric disorders. Therefore, investigating if there was an increase in new-onset psychiatric disorders is of great importance as it has long-lasting implications for patients, the health system and the economy (24, 25). Only a few studies have sought to further examine psychiatric diagnoses within the COVID-19 period in terms of chronic and new-onset diagnoses.

Most studies on new-onset psychiatric disorders during the COVID-19 pandemic focus on post-COVID-19 psychiatric disorders. There is increasing evidence that psychiatric disorders, such as depression (26–28), psychosis (29–32) and anxiety disorders (27, 28) frequently occur following a COVID-19 infection, often also as new-onset diagnoses (31, 33–35). Pathophysiologically, an immune response (cytokine storm) to an initial infection or a direct viral infection of the central nervous system might be the cause (27, 28, 31, 36).

In the current study, however, we are not focusing on the direct sequelae of COVID-19 infections but the indirect effect of the pandemic and its impact on new-onset psychiatric disorders. The indirect effect could result from fear of COVID-19 infection (37, 38), social isolation during lockdown (39, 40), loss of daily routines and financial insecurity (40). Changes within the medical care system such as reduced outpatient psychiatric and psychotherapeutic care, which was seen particularly during the first lockdowns (41–44) might also have led to an increase in new-onset psychiatric disorders.

Studies on new-onset psychiatric disorders through the indirect effect of the pandemic are scarce. An online survey from Italy showed that 16.0% of participants during the first wave and 18.6% of participants during the second wave of COVID-19 met the criteria for at least one new-onset psychiatric disorder, suggesting an increase compared to pre-pandemic times (45). A study of 850 individuals attending a pED during the first wave of the pandemic in Hannover, Germany assessed more treatment-naive patients with neurotic, stress-related, and somatoform disorders, than in the comparison time period 1 year earlier. The authors argue that this may point toward an increase in the new-onset of these disorders (46). A longitudinal comparative study from Israel showed a significant increase of 38.0% of new-onset psychosis or mania in pED presentations during the first wave (March–April 2020) (47). A study from New York found an increase in new-onset psychiatric disorders during the first wave in children and adolescents but not in adults (32).

To assess the indirect effect of the COVID-19 pandemic on new-onset of psychiatric diseases, this study focuses on the number of psychiatric new-onset diagnoses in pED presentations during the second wave of the pandemic compared to pre-pandemic times. We focused on diagnostic subgroups and, based on research from early phases of the pandemic, defined risk groups by age, gender, homelessness (11), attending in police custody (48) and familial relationship (7, 32, 49) that might be especially vulnerable. We investigated all records from patients of one major pED in Berlin, Germany during the second wave of COVID-19 to validate for new-onset diagnoses.

## 2. Materials and methods

### 2.1. Study design

This study was approved by the local ethics committee (Charité Universitätsmedizin, Berlin; number of approval: EA 110/20). We conducted a retrospective chart review comparing all presentations at an academic psychiatric emergency department (pED) in Berlin (St. Hedwig Hospital) during the second wave of the COVID-19 pandemic (9/15/2020–3/1/2021 = “COVID-19-period”) with all pED presentations of the same time period 1 year earlier as a baseline (“control period”).

The psychiatric department of Charité Universitätsmedizin Berlin at St. Hedwig Hospital (SHK) is responsible for providing psychiatric emergency care to the approx. 327,000 citizens of the districts Tiergarten, Wedding and Moabit. It consists of one emergency admission and seven psychiatric care units for inpatient treatment. Patients living in other districts of Berlin are usually redirected to the psychiatric clinic of their district when inpatient treatment is required.

We decided to study the second wave as it has been less studied than the first, while being more than twice as long and providing an opportunity to explore the effects of the implementation of a lockdown. The beginning of the second wave in Berlin (9/15/2020) is defined by a continuously rising 7-day-incident measure, the number of COVID-19 cases in the last 7 days per 100,000 citizens (50). The end (3/1/2021) is marked by the beginning of the relaxation of the COVID-19 policy [e.g., reopening of hairdressers (51)]. On December 16th 2020 a resolution for a “hard lockdown” came into force (52, 53). Private gatherings were limited to a maximum of five adults, the retail and gastronomic sector had to close with some exceptions, schools were closed and drinking alcohol in public spaces was forbidden. Clinical routine chart data documented in ORBIS^®^, the digital hospital software, from all patients from both time periods was extracted, including the pED cases and the cases with inpatient treatment.

Cases were excluded if they had duplicate clinical records, if the patient left without being seen by medical staff and if they did not have a psychiatric diagnosis according to the International Classification of Diseases version 10, Clinical Modification (ICD-10-GM-2022). Further exclusion criteria were day therapy cases, as admissions to the day therapy unit were restricted as part of the measures to reduce the spread of COVID-19. Scheduled inpatient admissions (not via the pED) were also excluded. This concerns mainly scheduled detoxifications. In the case of repetitive presentations of patients within one time period, we decided to include only the first attendance (S1).

This study refers to the new-onset of the main diagnosis only, as it can be considered to be the most reliably diagnosed across different psychiatrists (54) and to have the biggest impact on the patient. Main diagnoses were defined as new-onset if they were first diagnosed in the current hospital attendance. These may be individuals who had never received a psychiatric diagnosis before or individuals who had a history of another psychiatric diagnosis. We only considered a main diagnosis as new-onset when this diagnosis did not appear in any previous records of the patient, either as a main or a secondary diagnosis. For first-time patients at SHK with no earlier psychiatric records available, patients’ description of events and medical history obtained in the pED were used to determine if the main diagnosis fulfilled the criteria of new-onset or not.

Main diagnoses were grouped into nine subgroups: substance use disorders (without nicotine dependence/harmful use), depressive disorders, schizophrenia and psychotic disorders, anxiety disorders, trauma and stress-related disorders, other neurotic disorders, personality disorders, organic mental disorders and bipolar and manic disorders (S2).

Variables of particular interest were “homelessness” i.e., individuals with no shelter or who are staying in homeless shelters, “familial relationship,” defined as people that are in a relationship or have children; and “attendance in police custody” meaning people referred by the police to the pED.

### 2.2. Statistical analysis

Descriptive statistical analyses were performed to assess differences between new-onset of diagnoses during the second wave of the COVID-19 pandemic and the control period. Since the metric variable “age” was not normally distributed, the median is reported. Comparison of medians between the time periods was performed using the Mann–Whitney-U-Test. For all other categorical variables, absolute numbers and percentages are reported and compared using the Chi^2^ test. The value of p for statistical significance was set to *p* < 0.05 except for the diagnostic subgroups. For these analyses, we applied a Bonferroni correction for multiple testing to control the occurrence of false positives to the significance levels as follows: *p* = 0.05/9 = 0.0056. The logistic regression model was conducted to explore potential influence factors on new-onset diagnoses.

We assessed for diagnostic subgroups and risk factors found in the literature and based on our hypothesis, limiting ourselves to those that were well documented in our primary data: age, gender, homelessness, attending in police custody, familial relationships and time period. As an outcome variable, we chose new-onset vs. chronic. Results from the regression models are presented as odds ratios (OR) with 95% confidence intervals (95%CI) and are tested for significance using the Wald-Chi^2^ tests.

Statistical analyses were performed using the SPSS statistical package, version 27.0, IBM Corporation (2020). The cross correlation was created with the tseries package (55) in R 4.1.2. Tables were created using MS Excel 365, Microsoft Corporation (2020).

## 3. Results

A total of *n* = 4,010 records (patients *n* = 2,624) were documented during the two observed time periods (COVID-19-period: *n* = 1986; patients *n* = 1,312, control period: *n* = 2024; patients *n* = 1,312). After applying exclusion criteria, a total of n = 2,619 records (patients *n* = 2,445) were included in our analysis (COVID-period: *n* = 1,249, control period: *n* = 1,370; Table 1). 174 patients presented to the pED in both time periods. Eleven patients during the COVID-19-period were tested positive for COVID-19. Four patients reported a previous COVID-19 infection. For a detailed description of the demographic and clinical characteristics of all patients presenting to the pED, see Table 1.

**Table 1:** Clinical and demographic characteristics of Psychiatric emergency department (pED) presentations.

		Control period cases (% of all cases in period)	COVID-19-period cases (% of all cases in period)	Difference***%***	*p*-value
	N total number of cases	1,370	1,249	−8.8%	–
	Mean cases per week	57.1	52.0	−8.9%	**<0.001**
	Tested positive for COVID-19	–	11	–	-–
	Post COVID-19 infection	–	4	–	–
Attendance/ admission	Attendances in police custody	202 (14.7%)	237 (19.0%)	17.3%	**0.004**
	Involuntary admission	113 (8.2%)	135 (10.8%)	19.5%	**0.025**
	Hospital admission	631 (46.1%)	561 (44.9%)	−11.1%	0.558
Sociodemographic risk factors	Median age	39 years	39 years	–	0.41
	Age range	18–97	18–99	–	–
	N females	555 (40.5%)	529 (42.4%)	−4.7%	0.33
	Homelessness	168 (12.3%)	167 (13.4%)	−0.6%	0.404
	Job loss	16 (1.2%)	54 (4.3%)	237.5%	**<0.001**
	Familial relationship	498 (36.4%)	404 (32.3%)	−18.9%	**0.031**
	Living alone	281 (20.5%)	403 (32.3%)	43.4%	**<0.001**
	Conflicts	178 (13.0%)	190 (15.2%)	6.7%	0.103
	Unsafe residential status	35 (2.6%)	33 (2.6%)	−5.7%	0.892
Clinical symptoms	Signs of delusion	298 (21.1%)	313 (25.1%)	5.0%	**0.016**
	Obsessive thoughts	30 (2.2%)	23 (1.8%)	−23.3%	0.391
	Aggressive behavior toward others	141 (10.3%)	128 (10.2%)	−9.2%	0.962
Suicidality	Suicidal thoughts	357 (26.1%)	321 (25.7%)	−10.1%	0.464
	Suicidal plans	152 (11.1%)	143 (11.4%)	−5.9%	0.854
	Suicide attempts	54 (4.0%)	63 (5.1%)	16.7%	0.177

Comparison of demographic and clinical characteristics of pED presentations in corresponding time periods of the control period (on the left) and the COVID-period (on the right). The difference is the change of case numbers in the COVID-period compared to the corresponding control period in percentages. *p*-values (bold = significant to a level of *p* ≤ 0.05) are derived from chi^2^-tests, except for “median age,” which were tested using the Mann–Whitney-U-test. “Covid-19 positive” includes all patients tested at admission or during hospital treatment.

### 3.1. New-onset diagnoses and 7-day incidence rate

**Figure 1:**
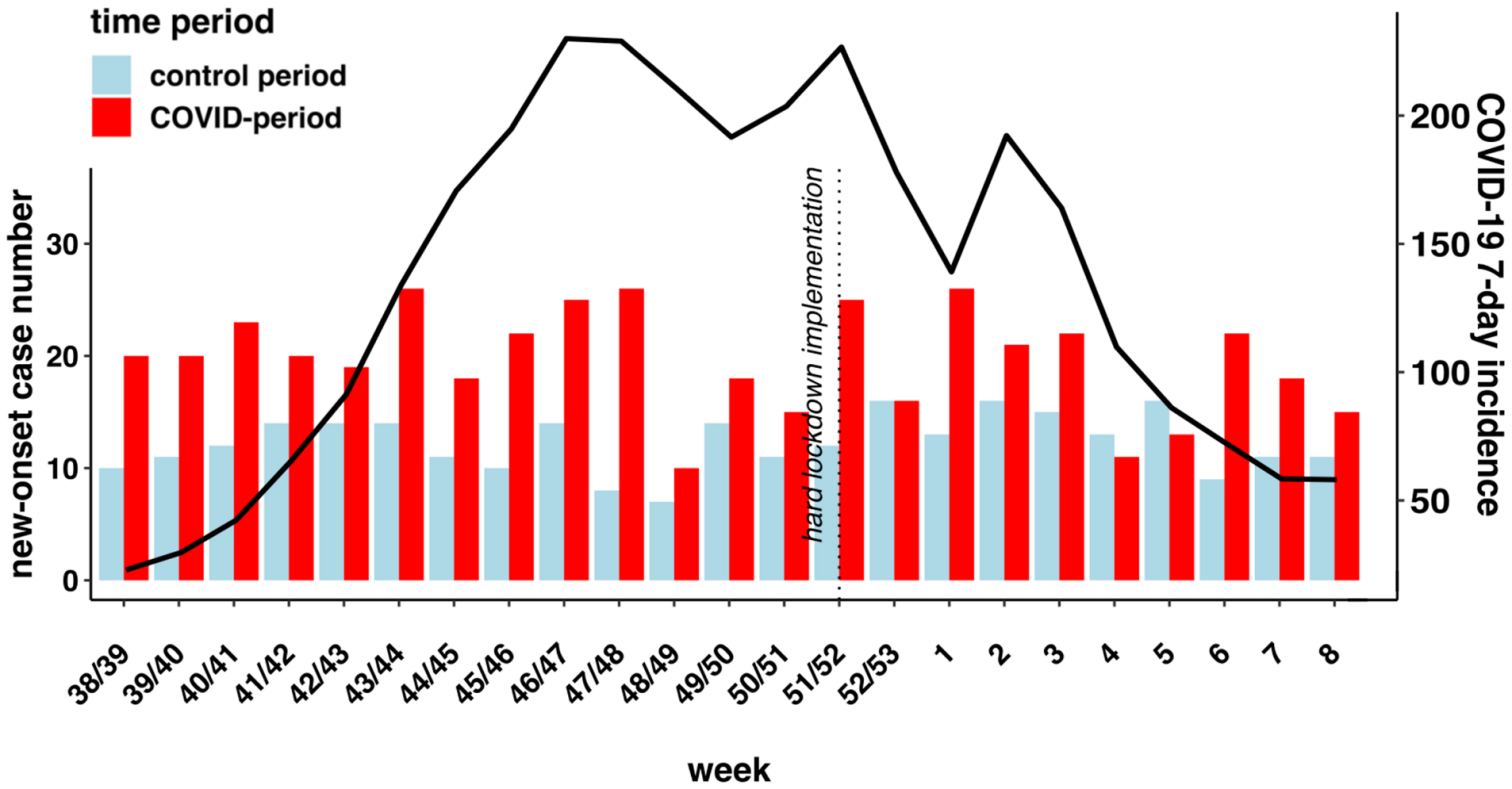
Displays weekly new-onset diagnoses in absolute numbers in the COVID-19-period (red bars) and control period (blue bars) and weekly 7-days incidence in Berlin (red line) in the calendar weeks 38-8 2019/2020 (control period) and 39-8 2020/2021 (COVID-19-period). Weeks 9 in both periods and 38 in the COVID-period are not displayed, as the observation periods did not include complete weeks. The vertical line indicates the enforcement of hard lockdown measures in Germany (12/16/2020). Data on Covid-19 incidence from “Landesamt für Gesundheit und Soziales”: https://www.berlin.de/lageso/gesundheit/infektionskrankheiten/corona/tabelle-indikatoren-gesamtuebersicht/; Abbreviations used: pED = psychiatric emergency department. Raw data on weekly new-onset presentations is shown in Supplementary data (S3).

The weekly number of cases with new-onset diagnoses during both observation periods is presented in Figure 1 along with the timeline of the 7-day incidence of COVID-19 cases in Berlin, Germany. The average weekly number of new-onset diagnoses was 59.3% higher during the COVID-19-period compared to the control period (Table 2). The range of new-onset cases was between 7 and 16 per day in the control period and 10–26 in the COVID-19-period without any significant peaks.

**Table 2:** New-onset vs. permanent diagnosis.

		Control period cases (% of all cases in period)	COVID-19-period cases (% of all cases in period)	Difference***%***	*p*-value
**Total case**	Mean new-onset cases per week	12.3	19.6	59.3%	**<0.001**
	New-onset of main diagnosis	295 (21.5%)	471 (37.7%)	59.7%	**<0.001**
	Permanent diagnosis	1,062 (77.5%)	764 (61.2%)	−28.1%	
	No information available	13 (0.9%)	14 (1.1%)	7.7%	
**Diagnostic subgroups**
	**Substance use disorders**				**<0.001**
	New-onset	40 (10.4%)	117 (32.4%)	192.5%	
	Permanent	335 (87.5%)	230 (63.7%)	−31.3%	
	No information available	8 (2.1%)	14 (3.9%)	75.0%	
	Total	383	361	−5.7%	
	**Depressive disorders**				**<0.001**
	New-onset	38 (20.9%)	82 (49.4%)	115.8%	
	Permanent	144 (79.1%)	84 (50.6%)	−41.7%	
	Total	182	166	−8.8%	
	**Schizophrenia spectrum and psychotic disorders**				**<0.001**
	New-onset	30 (10.2%)	64 (23.2%)	113.3%	
	Permanent	261 (88.8%)	212 (76.8%)	−18.8%	
	No information available	3 (1.0%)	0		
	Total	294	276	−6.1%	
	**Anxiety disorders**				**<0.001**
	New-onset	22 (28.2%)	36 (58.1%)	63.6%	
	Permanent	55 (70.5%)	26 (41.9%)	−52.7%	
	No information available	1 (1.3%)	0		
	Total	78	62	−20.5%	
	**Trauma and stressor-related disorders**				0.098
	New-onset	117 (73.1%)	96 (82.1%)	−17.9%	
	Permanent	42 (26.3%)	21 (17.9%)	−50.0%	
	No information available	1 (0.6%)	0		
	Total	160	117	−26.9%	
	**Other neurotic disorders**				0.446
	New-onset	7 (36.8%)	13 (48.1%)	85.7%	
	Permanent	12 (63.2%)	14 (51.9%)	16.7%	
	Total	19	27	42.1%	
	**Personality disorders**				**0.044**
	New-onset	6 (6.8%)	14 (16.7%)	133.3%	
	Permanent	82 (93.2%)	70 (83.3%)	−14.6%	
	Total	88	84	−4.5%	
	**Organic mental disorders**				0.100
	New-onset	25 (32.5%)	32 (45.7%)	28.0%	
	Permanent	52 (67.5%)	38 (54.3%)	−26.9%	
	Total	77	70	−9.1%	
	**Bipolar and manic disorders**				0.520
	New-onset	7 (11.3%)	9 (15.3%)	28.6%	
	Permanent	55 (88.7%)	50 (84.7%)	−9.1%	
Total	62	59	−4.8%	

Comparison of number of new-onset and permanent diagnosis in total cases and in diagnostic subgroups in corresponding time periods of the control period (on the left) and the COVID-period (on the right). The difference is the change of case numbers in the COVID-period compared to the corresponding control period in percentages. *p*-values (bold = significant to a level of *p* ≤ 0.05; except for diagnostic subgroups *p* = 0.0056 after Bonferroni correction) are derived from chi^2^-tests.

To test whether the 7-day incidence of COVID-19 cases predicts diagnosis significance at different lagged time points, we implemented a cross-correlation, which measures the degree of correlation between a time series and another time series lagged at different time points. Correlations ranged from (−0.295; 0.249) none of them reached significance (S4). Thus, the lockdown on 16th of December was not accompanied by effects on the number of new-onset diagnoses.

### 3.2. Prevalence of new-onset of psychiatric diagnoses during the COVID-19-period compared to the control period

There was a total of 295 (21.5%) cases with a new-onset of the main diagnosis in the control period and 471 (37.7%) of new-onset diagnoses during the COVID-19-period, indicating an increase of 59.7% in new-onset diagnoses during the COVID-19-period. Three patients with new-onset psychiatric diseases tested positive for COVID-19, and four had a positive history of COVID-19 infection (Table 3). The number of new-onset of substance use disorders (20.5% of new-onset cases in the COVID-19-period) was higher by 192.5% (*p* < 0.001), for depressive disorders (15.7% of new-onset cases in the COVID-19-period) the number was higher by 115.8% (*p* < 0.001), for schizophrenia spectrum and psychotic disorders (12.3% of new-onset cases in the COVID-19-period) the number was higher by 113.3% (*p* < 0.001) and for anxiety disorders (7.6% of new-onset cases in the COVID-19-period) the number was higher by 63.6% (*p* < 0.001) in comparison to the new-onset of these diagnostic subgroups during the control period. For all other diagnostic subgroups, no statistically significant differences in new-onset diagnoses were found.

**Table 3:** Characterization of pED presentations with a new-onset main diagnosis.

		Control period cases (% of all new-onset cases)	COVID-19-period cases (% of all new-onset cases)	Difference %	*p*-value
	N (% of total cases)	295 (21.5%)	471 (37.7%)	59.7%	
	tested positive for COVID-19	–	3	–	–
	post COVID-19 infection	–	4	–	–
Attendance/admission	attendance in police custody (%)	46 (15.6%)	114 (24.2%)	147.8%	**0.004**
	involuntary admission (%)	23 (7.8%)	59 (12.5%)	156.5%	**0.039**
	hospital admission (%)	102 (34.6%)	198 (42.0%)	94.1%	**0.040**
Sociodemographic risk factors	median age	34	35	2.9%	0.628
	age range	18–97	18–99	–	–
	n females (%)	132 (44.7%)	218 (46.3%)	65.2%	0.677
	homelessness (%)	23 (7.8%)	49 (10.4%)	113.0%	0.229
	job loss (%)	6 (2.0%)	23 (4.9%)	283.3%	**0.046**
	familial relationship (%)	133 (45.1%)	177 (37.6%)	33.1%	**0.039**
	living alone (%)	53 (18.0%)	160 (34.0%)	201.9%	**0.000**
	conflicts (%)	57 (19.3%)	92 (19.5%)	61.4%	0.943
	unsafe residential status (%)	12 (4.1%)	25 (5.3%)	108.3%	0.420
Clinical symptoms	signs of delusion (%)	58 (19.7%)	111 (23.6%)	91.4%	0.205
	obsessive thoughts (%)	7 (2.4%)	11 (2.3%)	57.1%	0.915
	aggressive behavior toward others (%)	23 (7.8%)	52 (11.0%)	126.1%	0.146
Suicidality	Suicidal thoughts (%)	71 (24.1%)	122 (25.9%)	71.8%	0.704
	Suicidal plans (%)	39 (13.2%)	55 (11.7%)	41.0%	0.480
	Suicide attempts (%)	18 (6.1%)	30 (6.4%)	66.7%	0.895

“Difference” is the relative change of absolute numbers in the “control period” compared to the “COVID-period’ in percentage values. *p*-values are resulting from chi^2^-tests, except for median age, which were tested using the Mann–Whitney-U-test. *p*-values (bold = significant to a level of *p* ≤ 0.05).

Patients with new-onset diagnoses during the COVID-19-period compared to the control period were more often attending in police custody (*p* = 0.004; diff. +147.8%), had in absolute numbers more often, but proportionally less often, a familial relationship (*p* = 0.039; diff. +33.1%), were more often admitted to the hospital (*p* = 0.040; diff. +94.1%), more often involuntarily (*p* = 0.039; diff. +156.5%), had more often experienced job loss (*p* = 0.046; diff. +283.3%) and were more often living alone (*p* < 0.001; diff: +201.9%; Table 3).

### 3.3. Time-dependent (during the COVID-19-period) factors associated with new-onset of diagnoses

Patients diagnosed with substance use disorders (*p* < 0.001; OR 3.28; 95% CI 1.97–5.46), depression (*p* < 0.001; OR 3.28; 95% CI 1.87–5.74) or anxiety disorders (*p* = 0.009; OR 2.83; 95% CI 1.30–6.15) had around three times higher risk of having a new-onset during the COVID-19-period (Table 4). Patients with diagnoses of schizophrenia spectrum and psychotic disorders (*p* = 0.034; OR 1.84; 95% CI 1.05–3.22) were also more susceptible to having a new-onset of their disease.

**Table 4:** Binominal logistic regression analysis.

		Exp (B)	95% CI lower	95% CI upper	*p-v*alue
Risk factors and diagnostic subgroups (time independent)	Age	0.981	0.973	0.989	**<0.001**
	Gender	0.895	0.669	1.197	0.454
	In police custody	1.299	0.872	1.934	0.198
	Familial relationship	1.561	1.168	2.087	**0.003**
	Homelessness	0.736	0.447	1.211	0.227
	COVID-19-period	0.955	0.522	1.746	0.880
	Substance use disorders	0.182	0.123	0.271	**<0.001**
	Depressive disorders	0.392	0.258	0.595	**<0.001**
	Schizophrenia spectrum and psychotic disorders	0.185	0.120	0.286	**<0.001**
	Anxiety disorders	0.576	0.333	0.995	**0.048**
Interactions (time dependent)	COVID-19-period by age	1.003	0.992	1.015	0.546
	COVID-19-period by gender	1.275	0.865	1.878	0.220
	COVID-19-period by in police custody	1.811	1.092	3.003	**0.021**
	COVID-19-period by familial relationship	0.877	0.592	1.299	0.513
	COVID-19-period by homelessness	1.099	0.584	2.065	0.770
	COVID-19-period by substance use disorders	3.279	1.969	5.461	**<0.001**
	COVID-19-period by depressive disorders	3.276	1.869	5.743	**<0.001**
	COVID-19-period by schizophrenia spectrum and psychotic disorders	1.836	1.045	3.224	**0.034**
	COVID-19-period by anxiety disorders	2.825	1.297	6.150	**0.009**

Results of logistic regression analysis on factors potentially associated with new-onset diagnosis. Bold print indicates statistical significance at 0.05 level.

Individuals who came to the pED in police custody during the COVID-19-period were 1.81 times (*p* = 0.021; 95% CI: 1.09–3.00) more likely to present with new-onset diagnoses than their counterparts attending the pED without police. Age, gender, being homeless or having a familial relationship was not associated with the risk of having a new-onset diagnosis during the COVID-19-period.

### 3.4. Time-independent factors associated with new-onset of diagnoses (logistic regression analysis)

Older age predicted a lower risk of having a new-onset of diagnoses, independently of the time period (*p* < 0.001; OR 0.98; 9% CI 0.97–0.99). Having a familial relationship (partnership or children) was associated with a higher risk for a new-onset diagnosis (*p* = 0.003; OR 1.56; 95% CI 1.17–2.09; Table 4). The diagnostic subgroups substance use disorders (*p* < 0.001; OR 0.18; 95% CI 0.12–0.27), depression (*p* < 0.001; OR 0.39; 95% CI 0.26–0.60), schizophrenia spectrum and psychotic disorders (*p* < 0.001; OR 0.19; 95% CI 0.12–0.29) and anxiety disorders (*p* = 0.048; OR 0.58; 95% CI 0.33–1.00) predicted a significantly lower risk for a new-onset diagnosis.

## 4. Discussion

This study investigated the incidence of new-onset psychiatric diagnoses as well as further contributing factors among patients presenting to a major pED in Berlin during the second wave of the COVID-19 pandemic, based on a retrospective cross-sectional study.

The current study shows an approximately consistent number of cases in both time periods, in contrast to most previous studies showing a decrease in psychiatric presentations in the early COVID-19-periods (10, 11, 13, 56, 57). This is likely due to local differences and the later observation period in comparison in the current study.

A higher number of patients with new-onset diagnoses presented to the pED during the COVID-19-period compared to the control period. During the COVID-19-period, presentations of new-onset substance use disorders, depressive disorders, schizophrenia spectrum and psychotic disorders, and anxiety disorders were more frequent than during the control period. Furthermore, the presence of these diagnostic subgroups and being referred by the police, predicted a pED presentation with new-onset diagnosis during the COVID-19-period in our logistic regression analysis.

### 4.1. Substance use disorders

The new-onset of substance use disorders increased by 192.5% during the COVID-19-period and substance use disorders were more than three times more likely to be new-onset (*p* < 0.001; OR 3.28; 95% CI 1.97–5.46). Most studies report an increase of substance use disorders in the general population during the COVID-19-period (58, 59). However, among pED presentations, the number of patients with substance use disorders decreased at the beginning of the pandemic (60, 61). This is also true for our sample: the absolute number of pED presentations with substance use disorders decreased by 5.7% (Table 1) while new-onsets increased.

In a sub-analysis (S5), we see that the most important factor in the increase in new-onset substance use disorders in our sample is the increase in pED presentations with acute alcohol intoxication (33.3% of new-onset substance use disorders during the COVID-19-period). This is in line with the literature, showing increases in alcohol consumption at the beginning of the pandemic (62, 63). Also, differences in alcohol use patterns are reported, with more binge/heavy drinking during a lockdown and an increase in alcohol-related emergencies (64).

Low-threshold services such as group meetings for people dealing with addictions were not taking place regularly anymore (65), which might have driven patients to attend the pED. Possibly, there was a shift from consumption in social situations to consumption at home, due to restrictions (4). Conceivably, this shifted the perception of users regarding their consumption from being legitimized by social activities to being pathological when alone. Loneliness, which is described as a pathogenetic factor during the COVID-19-period (40, 66), might have also triggered more heavy consumption patterns. Finally, supply shortages of drugs, due to travel restrictions, may have driven people to source supplies from unfamiliar providers, increasing the risk of exposure to contaminated substances (67).

### 4.2. Depressive disorders

Depressive disorders increased by 115.8% during the COVID-19-period, in comparison to the control time. Patients diagnosed with depression had an approximately three times higher risk of having a new-onset of the disorder (*p* < 0.001). Many studies from the beginning of the pandemic report an increase in depressive symptoms without differing between new-onset and chronic (6, 8, 9, 38, 68–71).

An online study from Italy found an increase in new-onset major depressive disorders in the first and second wave among the general population (72). A longitudinal survey among university students in Japan found that 11.8% had a new-onset of depressive symptoms, supporting our findings (73).

During the pandemic, known risk factors for depression such as social isolation/loneliness (40), job loss (74, 75) and financial insecurity were on the rise. This can also be seen in our sample: job loss and living alone were significantly more prevalent in the COVID-19-period than in the control period (Table 1). These circumstances may have led to an increase in new-onset depressions. In addition, patients with new symptoms of depression, who would normally have consulted psychiatric practices or other outpatient mental health services, instead turned to the pED (42). This mechanism likely applies to the other diagnostic subgroups as well.

Depression has the highest lifetime prevalence among psychiatric diagnoses (76). Possibly, the pandemic precipitated the onset of depression in vulnerable patients.

### 4.3. Schizophrenia spectrum and psychotic disorders

There was a significant increase in the prevalence of new-onset schizophrenia spectrum and psychotic disorders. Furthermore, this diagnostic subgroup was a predictor for presentations with a new-onset diagnosis during the COVID-19-period (*p* = 0.034). This finding is in line with prior studies that saw the number of patients with psychotic disorders rising during the pandemic, both directly via neuropsychiatric sequelae after SARS-CoV-2 infection (36, 77, 78) and indirectly (10, 14, 79, 80). In line with this, there were also significantly more cases with signs of delusion in the COVID-19-period than the control period (Table 1).

The already mentioned Israeli study shows that the increase in pED presentations with psychotic disorders and mania correlated highly with lockdown measures and not with national incidence rates (47). Their new-onset cases increased by 45.5% compared to 2019. In our sample, the increase was 113.3%. In their study, the difference in the overall proportion of new-onset diagnoses (psychosis and mania) was 5.5% in 2019 and 8% in 2020 of all diagnoses with psychosis and mania. In the current study, the rate of new-onset psychotic disorders is 10.2% in 2019 and 23.2% in 2020. In another study, also conducted in Israel, a decrease in the incidence rate for schizophrenia was found for the period from March 2020 to February 2021 compared with the years before (81). However, the decrease may rather reflect reduced utilization of medical services by chronic schizophrenic patients and is no clear evidence against a rise in new-onset cases.

### 4.4. Anxiety disorders

While fewer patients with anxiety disorders presented to the pED in general, patients with anxiety disorders showed an increase of 63.6% of new-onset during the COVID-19-period and had a higher risk for a new-onset during the COVID-19-period. These results are in line with other studies showing an increase in symptoms of anxiety in the general population (6, 8, 9) as well as in pED presentations (17, 32). One study showed an increase of 35% in pED presentations with anxiety disorders (17), although no distinction was made between new-onset and chronic diagnoses. The study from a pED in New York found an increase in anxiety disorders in adults of 200% during the COVID-19-pandemic. Here, however, the results are probably not representative due to the insufficient sample size of a total of 16 patients with anxiety disorders (32).

A Canadian study found that fewer young patients presented to primary care with a new episode of anxiety during the first wave, albeit incidence rates were higher during the second wave than before the pandemic. Older adults were found to have higher incidence rates of anxiety disorders in both waves than before the pandemic (82). Age was not found to be a predictor of new-onset in our study. This suggests local differences in risk factors for new-onset diagnoses.

Reasons for this increase are not fully understood yet. There might be more fear in general because of the pandemic, such as fear of a COVID-19 infection (37) as well as more social fears due to social distancing (83).

### 4.5. Police custody

While there was a decline in the absolute number of patients attending police custody, there was an increase in the relative attendance in police custody. Significantly, a high proportion of patients with a new-onset of a psychiatric disorder during the COVID-19-period attended police custody.

Other studies also show higher proportions of police referrals (48, 84, 85) but do not report if these patients had chronic or new-onset diseases. A Canadian study indicated a drastic increase of emergency police calls involving persons with perceived mental illness, especially in the second wave (86).

In the current study “attendance in police custody” was a predictor of new-onset diagnoses during the COVID-19-period, suggesting high acuity of these cases. In line with this, we found that hospital admissions and involuntary admissions did occur more often in new-onset diagnoses during the COVID-19-period than during the control period. This implies that the more severe cases were also the cases that were more likely to be newly diagnosed. People might have waited until it was too late to be able to go voluntarily to the hospital, for fear of getting infected with COVID-19, which might account for the increase in attendance in police custody. A study from London showed that patients were likely to experience a longer duration of symptoms before seeking help from mental health services during the COVID-19 pandemic (14).

### 4.6. New-onset diagnoses and 7-day incidence rate

We could not find a correlation between the number of new-onset diagnoses and the 7-day incidence of COVID-19 cases in Berlin (Figure 1). This is in line with a study from Israel showing no epidemiological evidence for a causal link between the number of COVID-19 cases and the increased ratio of new-onset psychosis and mania during the first and second wave of COVID-19 (47).

Our results do not show a concordant increase in pED presentations with new-onset psychiatric disorders along with the implementation of a hard lockdown (Figure 1). This is in contrast to the above-mentioned Israeli study, which did report a correlation between lockdown and an increase in new-onset of psychosis and mania (47). This finding may indicate different lockdown implications in different countries or different populations. In Turkey, for example, there is evidence even for improvement of mental health symptoms during the first lockdown in college students (87). Heterogeneities like this, stress the potential impact of local differences. In addition, differences in the extent of lockdown measures may be an explanatory factor (88).

### 4.7. A history of COVID-19 infection as a reason for the increase in psychiatric emergency department presentations with new-onset psychiatric disorders

If post-COVID-19 was the driving factor behind the increase in new-onset pED presentations, one would expect an increase in new-onset cases over time as the virus continued spreading. This was not the case in our sample. Furthermore, in the current study we report an increase in new-onset psychiatric disorders not only in depression disorders, anxiety disorders and schizophrenia spectrum and psychotic disorders, which are also reported as being caused by COVID-19 infections (26–32), but also in substance use disorders, which up until now, have not been linked to COVID-19 infections. In summary, we posit that the increases in new-onset psychiatric disorders reported in this study are for the most part not due to a prior COVID-19 infection but due to the indirect effect of the pandemic.

## 5. Strengths and limitations

This study is the first to systematically investigate the prevalence of new-onset psychiatric diagnoses in pED presentations during the second wave of COVID-19, together with associated risk factors. The current study covers a relatively long observation period with a comparably large number of assessed pED presentations. Indicators of mental health were based on clinical diagnoses rather than self-reports. In addition, our detailed clinician-led review of each case was based on thorough clinical documentation and gave detail to elucidate the changes during the COVID-19-period.

The following limitations need to be considered: the control data is limited to the previous year only. We cannot rule out the possibility that the control year had an unusual low ratio of new-onset diagnoses. Furthermore, the study only reflects mental health during a part of the pandemic and may thus miss rebound effects in post-lockdown periods or long-time effects. The study is based on clinical routine data which can differ in quality and extent which may introduce bias. We cannot completely rule out the possibility of an interrater bias. However, to limit this bias we implemented the following measures: consulting all available data and scheduling regular meetings to discuss pressing questions, resolving them in consensus.

A further limitation is that we only gathered information about patients presenting with new-onset diagnoses in a single-center psychiatric emergency department. Extrapolation of results should therefore be done with caution. Besides, taking into account all diagnostic subgroups, our study does not provide information on whether there was a complete new-onset of symptoms or whether a decompensation of prior “subthreshold” psychiatric symptoms has occurred.

Only very few patients presented with a COVID-19 infection or a history of COVID-19 infection. Asymptomatic infections and underreporting are likely.

## 6. Conclusion

Psychiatric emergency department presentations with new-onset diagnoses of substance use disorders, depressive disorders, schizophrenia spectrum and psychotic disorders and anxiety disorders strongly increased during the COVID-19-period. These diagnoses and attendance in police custody were predictors of new-onset diagnoses during the second wave of the pandemic.

The current study provides evidence suggesting that the underlying factors affecting these increases in new-onset diseases at this phase of the pandemic were generally not directly linked to COVID-19 infections, but rather to other indirect sequelae of the pandemic. The current study reports greater job loss, living alone, and a relative decrease in familial relationships in patients with new-onset psychiatric diseases in the COVID-19-period as compared to the control period. These factors might have contributed to the increase in new-onset psychiatric diseases. Further studies are needed to assess the respective effects and other potential pathogenic factors. However, to date it is evident that some pathogenic factors are man-made and unintended sequelae of strict lockdown policies. Therefore, our findings should be taken into account for future pandemic control policies.

## Data availability statement

The raw data supporting the conclusions of this article will be made available by the authors, without undue reservation.

## Ethics statement

The studies involving humans were approved by the local ethics committee (Charité Universitätsmedizin, Berlin; number of approval: EA 110/20). The studies were conducted in accordance with the local legislation and institutional requirements. Written informed consent for participation was not required from the participants or the participants’ legal guardians/next of kin in accordance with the national legislation and institutional requirements.

## Author contributions

TG and SG conceptualized this study. MA wrote the original draft, edited and critically revised the manuscript. TG, SG, MS-O, YK, and JM edited and critically revised the manuscript. MA and YK extracted data from clinical documentation records. MA conceptualized and executed statistical analyses together with JM. All authors contributed to the article and approved the submitted version.
