# Supplementary material for: Increase in presentations with new-onset psychiatric disorders in a psychiatric emergency department in Berlin, Germany during the second wave of the COVID-19 pandemic – a retrospective cross-sectional study
Source: Front Psychiatry. 2023 Oct 12;14:1240703. doi: 10.3389/fpsyt.2023.1240703 (PMC10613500; doi:10.3389/fpsyt.2023.1240703)
Supplement: Supplementary file 1 [file Data_Sheet_1.docx]

**S1 Flow Chart**

**N = 4634** cases during the 2 observation periods

**N = 4237** cases after exclusion criteria

**N = 4010** cases after merging cases

**N = 2619** cases included in analysis

**n = 34** cases with duplicate clinical records

**n = 276** cases left without being seen by medical/psychiatric staff

**n = 67** somatic cases

**n = 3** consultations in preparation of a planned hospital admission

**n = 6** day therapy cases (patients receive treatment in hospital but sleep in their own home)

**n = 10** cases with non-medical issues (e.g.: patients in need of shelter)

**n = 1** patient <18 years

**n = 227** cases merged:

**n = 151** cases with *0-3 days* interruption of hospital admission

**n = 48** cases with *4-7 days* interruption of hospital admission

**n = 28** visits of emergency department, in between two merged hospital admissions

**n = 1192** cases of re-attendance

**n = 199** elective cases

**S2 Composition of diagnostic subgroups**

Organic mental disorders (OMD): F00 – F09

Substance use disorders (SUD): F10 – F19

Not included: F17 nicotine/tobacco related substance use disorders

Schizophrenia and psychotic disorders (SPD): F20 – F29

Bipolar and manic disorders (BMD): F30 – F31

Depressive disorders (DD): F32 – F33

Anxiety disorders (AD): F40 – F41

Trauma and stressor-related disorders (TSD): F43.0, F43.1, F43.2, F43.8

Other neurotic disorders (ND): F42, F44, F45, F48

Personality disorders (PD): F60

**S4**

The figure above indicates the size of the prediction of new-onset diagnosis by 7-day incidence at different lag points. The correlations do not reach significance, as they do not cross the blue lines. Abbreviations used: ACF = autocorrelation function
